# Identifying Country-Level Risk Factors for the Spread of COVID-19 in Europe Using Machine Learning

**DOI:** 10.3390/v14030625

**Published:** 2022-03-17

**Authors:** Serafeim Moustakidis, Christos Kokkotis, Dimitrios Tsaopoulos, Petros Sfikakis, Sotirios Tsiodras, Vana Sypsa, Theoklis E. Zaoutis, Dimitrios Paraskevis

**Affiliations:** 1 AIDEAS OÜ, Narva mnt 5, 10117 Tallinn, Estonia; s.moustakidis@aideas.eu; 2 Department of Physical Education and Sport Science, Democritus University of Thrace, 69100 Komotini, Greece; ckokkoti@affil.duth.gr; 3 Center for Research and Technology Hellas, Institute for Bio-Economy & Agri-Technology, 38333 Volos, Greece; d.tsaopoulos@certh.gr; 4 Joint Rheumatology Program, First Department of Propaedeutic Internal Medicine, Medical School, National and Kapodistrian University of Athens, 11527 Athens, Greece; psfikakis@med.uoa.gr; 5 Fourth Department of Internal Medicine, Attikon Hospital, Medical School, National and Kapodistrian University of Athens, 11527 Athens, Greece; sotirios.tsiodras@gmail.com; 6 Department of Hygiene, Epidemiology and Medical Statistics, Medical School, National and Kapodistrian University of Athens, 11527 Athens, Greece; vsipsa@med.uoa.gr; 7 Second Department of Paediatrics, “P. & A. Kyriakou” Children’s Hospital, Medical School, National and Kapodistrian University of Athens, 11527 Athens, Greece; t.zaoutis@eody.gov.gr; 8 National Public Health Organization, 15123 Athens, Greece

**Keywords:** COVID-19, machine learning, data mining, explainability

## Abstract

Coronavirus disease 2019 (COVID-19) has resulted in approximately 5 million deaths around the world with unprecedented consequences in people’s daily routines and in the global economy. Despite vast increases in time and money spent on COVID-19-related research, there is still limited information about the factors at the country level that affected COVID-19 transmission and fatality in EU. The paper focuses on the identification of these risk factors using a machine learning (ML) predictive pipeline and an associated explainability analysis. To achieve this, a hybrid dataset was created employing publicly available sources comprising heterogeneous parameters from the majority of EU countries, e.g., mobility measures, policy responses, vaccinations, and demographics/generic country-level parameters. Data pre-processing and data exploration techniques were initially applied to normalize the available data and decrease the feature dimensionality of the data problem considered. Then, a linear ε-Support Vector Machine (ε-SVM) model was employed to implement the regression task of predicting the number of deaths for each one of the three first pandemic waves (with mean square error of 0.027 for wave 1 and less than 0.02 for waves 2 and 3). Post hoc explainability analysis was finally applied to uncover the rationale behind the decision-making mechanisms of the ML pipeline and thus enhance our understanding with respect to the contribution of the selected country-level parameters to the prediction of COVID-19 deaths in EU.

## 1. Introduction

COVID-19 is caused by SARS-CoV-2, which belongs to the beta-coronaviruses and is characterized by cough, shortness of breath, and fever, symptoms similar to the ones associated with the seasonal flu [1]. Disease severity is associated with underlying comorbidities and increasing age [2]. The worldwide spread of COVID-19 has caused unprecedented effects on people’s daily routines and the prosperity of the economy [3,4]. The most important consequences of this pandemic are the burden on human health, either with permanent or temporary health problems, but mainly the huge number of associated deaths despite the interventions by the world community [5,6,7]. Hence, from the beginning of the COVID-19 pandemic until today, there is a need to understand the mechanism and factors that govern this disease. Specifically, as of 24 January 2022, a total of 340,543,962 confirmed cases with 5,570,163 confirmed deaths have been reported around the world resulting from COVID-19 (access on 24 January 2022, https://covid19.who.int/).

An increasing understanding of COVID-19 spread patterns and disease severity may lead to the development and efficient implementation of new treatments, vaccines, and measures that reduce the risk of adverse outcomes. In this difficult task, it is necessary to combine big data, today’s extreme computing power capacity, and the advanced currently available artificial intelligence (AI) tools [8,9,10,11]. The literature review so far has shown an increasing integration of the above in order to understand and cope with the COVID-19 pandemic. Advanced AI tools (data mining techniques) have already provided knowledge and valid hidden patterns to cope with the difficult task of COVID-19 understanding [12,13,14,15]. Naseem et al. demonstrated the key role of AI tools in healthcare in low-middle-income countries (LMIC) [12]. Specifically, they showed the power of the use of AI in the field of the diagnosis, management, and treatment of COVID-19 patients. Moreover, Debnath et al. presented a study to highlight the utility of AI prediction tools on a multitude of clinical settings [13]. Kolozsvári et al. proposed an approach to predict the epidemic curve of COVID-19 using AI [15]. They used data from Johns Hopkins University and the World Health Organization from the first and second waves in combination with an ensemble-based system, which is based on the interconnection of several neural networks, but they did not provide identified features that shape the model output. Muhammad et al. proposed data mining ML models in order to predict the stability and recovery of the newly infected patients with the novel coronavirus (COVID-19) [16]. They developed models for the prediction of the infected patients’ recovery by using an epidemiological dataset of COVID-19 patients of South Korea. Furthermore, a prediction model for the incidence of COVID-19 in Iran was proposed in [17]. They offered a data mining approach in order to help health managers and policymakers to control an epidemic outbreak and to plan the health care resources. 

In another study, an ML algorithm was proposed to increase COVID-19 inpatient diagnostic capacity [18]. They retrospectively used epidemiological and clinical data (e.g., demographics, complete blood counts, and inflammatory markers) and tested seven well-known ML models that achieved excellent diagnostic metrics compared to PCR tests. Moreover, they presented the individual importance of the employed features. In addition, Prakash et al. performed an extensive analysis on a COVID-19 dataset and employed various ML models to examine age effects on COVID-19-related outcomes [19]. They presented the contribution of the features for each age subgroup. Malki et al. investigated the association between weather data and the COVID-19 pandemic using ML approaches for the prediction of the mortality rate [20]. Specifically, the ML models were employed to estimate the impact of weather variables in the COVID-19 pandemic. In contrast to the previous studies, Bastani et al. proposed a real-time system, which is called “Eva”, for targeted COVID-19 screening [21,22]. They used reinforcement learning and real-time data in order to identify asymptotically infected with COVID-19 travelers and to provide real-time information for decision making. The paper was cited as one of the best examples of data use in the context of the epidemic.

To the best of our knowledge and based on the aforementioned studies, there is still limited information about the factors at the country level that affected COVID-19 transmission and fatality in EU. To examine this, a hybrid dataset was created integrating heterogenous, publicly available data from different sources, such as mobility changes, policy responses, vaccinations, and generic parameters, e.g., demographics. Then, a ML pipeline was designed, implemented, and tested, with the ultimate objective to estimate the number of COVID-19 deaths using the aforementioned inputs for the first three pandemic waves. Explainability analysis was finally employed on the trained models to uncover the rationale behind the decision-making mechanisms of the ML models and enhance our understanding of the impact of each country-level parameter on the prediction output (total number of new deaths per 1,000,000 people for each wave). 

## 2. Materials and Methods

### 2.1. Dataset 

#### 2.1.1. Data Sources

In this study, we employed data from the databases “Our World in Data” (https://ourworldindata.org/coronavirus, access on 8 October 2021) [23,24] and “Google COVID-19 Community Mobility Reports” (https://www.google.com/covid19/mobility/, access on 8 October 2021). Our dataset includes all the historical data of the COVID-19 pandemic up to 3 October 2021. These data include the first three waves of the pandemic for 33 countries of the European Continent. The duration of each wave was determined according to the average number of cases per 7 days. The data are divided into nine categories (Table 1) and have the aim to provide information about confirmed cases, hospitalizations, deaths, vaccinations, mobility, and testing as well as other generic variables at combating COVID-19. 

#### 2.1.2. Feature Extraction

**Figure 1:**
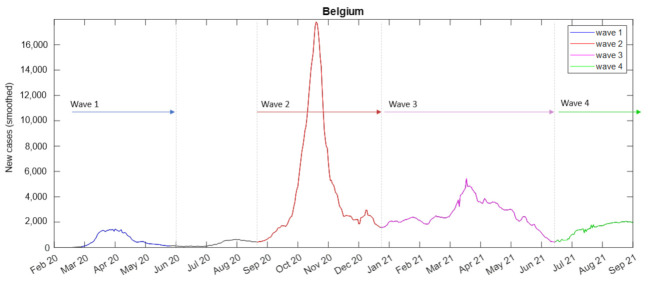
COVID-19 waves for Belgium.

Forty country-level parameters coming from all the aforementioned feature categories were extracted for each one of the three first pandemic waves. First of all, the three first pandemic waves were defined using the number of new daily cases (smoothed) as the main criterion. An indicative example of the waves’ determination is given in Figure 1 for Belgium, in which the waves 1–4 are depicted with blue, red, magenta, and green colors, respectively. The three first waves were considered in our paper since wave 4 was still ongoing in the majority of the EU countries at the time of the analysis.

Table 2 cites the main characteristics of the extracted features. Specifically, six mobility measures, two policy responses (stringency index and response time), nine metrics related to the number of vaccinations, and fifteen generic country-level parameters were included in the analysis. The mean value over the duration of the current wave was calculated for the mobility parameters, and the same calculation was applied to the stringency index. The response time was defined as the number of days needed to reduce the mobility measures by 30% with respect to pre-pandemic levels. For parameters representing total numbers, for example, total number of fully vaccinated people or total number of cases/deaths, the last valid value of the wave was extracted, whereas for parameters representing daily numbers (e.g., new cases per day or new vaccinations per day), the mean value over the duration of the wave was calculated. For the cases of wave 2 and 3, a subset of features was also considered from the previous waves (wave 1 and 2, respectively) including policy responses (features F7–8), vaccination status (F14–16), and the number of cases, deaths, hospitalizations, and positivity of the previous wave (F33–40). These extra parameters were included assuming that the previous status of the pandemic spread was expected to play a significant role in predicting the current spread.

### 2.2. Proposed Methodolgy 

The proposed AI methodology consists of five processing steps: (i) data pre-processing to normalize the extracted features and handle missing values, (ii) feature exploration to reduce the dimensionality of the initial feature space and identify a subset of important risk factors, (iii) learning phase utilizing a linear regression model, (iv) validation of the regression results using 10 fold cross validation (10KFCV), and (v) explainability analysis to quantify the impact of the selected risk factors on the produced decisions. A detailed presentation of the processing steps is given in the following subsections.

#### 2.2.1. Data Pre-Processing 

Mode imputation was employed to handle categorical and continuous missing values [25]. In our study, data were normalized to (0, 1) to build a common basis for the feature exploration and learning algorithms that follow [26]. 

#### 2.2.2. Feature Exploration 

Feature dimensionality reduction is a crucial task in our problem given the small number of samples (number of EU countries) and the relatively high number of features considered (40). To handle this challenge, a random feature exploration exercise was performed in which 5000 different feature subsets of varying dimensionality were tried out per wave. The proposed methodology was applied on each one of the 5000 feature subsets, and the best one (that minimizes the 10KFCV mean square error) was finally selected. This feature exploration was performed for each one of the three waves. 

#### 2.2.3. Regression 

**Figure 2:**
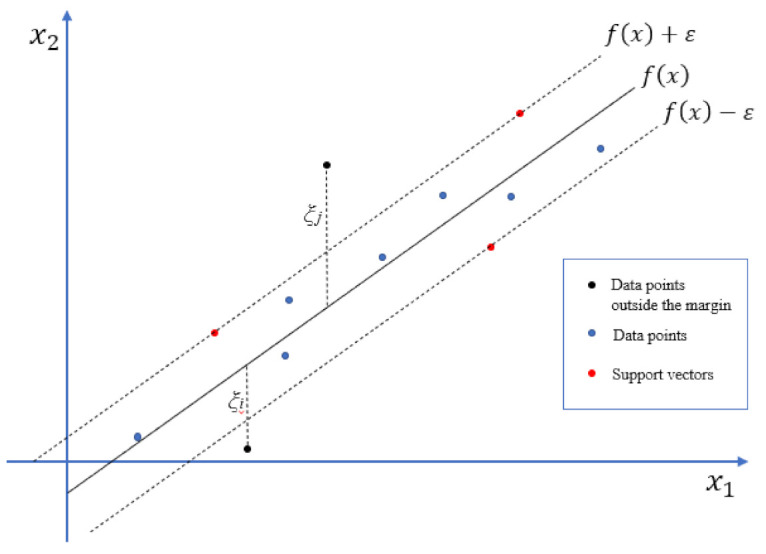
Model graph for support vector regression.

Following the feature selection task, a linear regression model was applied on the selected features. The main objective of the deployed regression model is to predict the number of deaths per wave given the selected input parameters from the current wave and optionally from the previous one (in case they were selected for waves 2 and 3). Linear epsilon-insensitive SVM (ε-SVM) [27,28] (also known as L1 loss) was employed to implement the regression task in which the goal is to find a function f(***x***):

(1) $$f(x)=x\prime \;w+b,\;x\;\in R^{n}$$

that deviates from *y* by a value no greater than ε for each training point ***x*** while being as flat as possible, where ***x*** is a multivariate set of *N* observations with observed response values *y*. To maximize flatness, f(***x***) needs also to minimize the norm value (**w**′ **w**), thus increasing the generalization of the model. Specifically, the objective of ε-SVM is to minimize the error rate and at the same time to fit the error within a certain margin, which is called ε-tube. A symmetrical loss function is used during the training, equally penalizing both high and low misestimates. Thus, a flexible tube of minimal radius is formulated around f(***x***) (the so-called margin) as seen in Figure 2. Data points outside the margin are penalized, whereas data points within the margin are the ones that do not receive any penalty. Such a loss function leads to a sparser decision rule representation, which comes with a number of advantages. Compared to conventional linear regression techniques, SVMs are effective in spaces of high dimensionality, especially when the number of features is comparable or even higher than the number of samples. SVM regression was employed in our study because it can work on small datasets of high dimensionality, as is in our test case, while keeping high accuracies with strong global search ability and optimization speed. Linear ε-SVM regression models were only considered here due to the small number of training samples. 

### 2.3. Validation and Explainability 

Ten-fold cross validation was employed to validate the performance of the proposed ML methodology. One of the main objectives of the present work is to examine how the different country-level risk factors contribute to the final prediction of deaths reported during each one of the three first pandemic waves. The linear ε-SVM regression model employed here forces the prediction to be a linear combination of features, and the effect of a feature j is actually quantified by the j-component of the weight (**w***_j_*). Therefore, the interpretation of the features in the linear regression model [29] were automated by using the following reasoning: Increasing the numerical feature **x***_j_* by one unit increases the estimated outcome y by **w***_j_* units when all the other features remain fixed. In the case of categorical features, changing **x***_j_* from another category to another increases the estimated outcome y by **w***_j_* units when all the other features remain fixed. 

## 3. Results

In this section, the overall predictive performance of the proposed ML pipeline is demonstrated for each one of the three pandemic waves. Specifically, each wave was treated separately as an individual data problem with its own input parameters (as they were defined in Section 2.1) and the total number of deaths per wave as the main output variable. Then, reference was made in the most important risk factors, as they were selected by the feature exploration technique (presented in Section 2.2.2), and finally, explainability analysis was employed to quantify the impact of these input parameters to the prediction of deaths, enhancing our understanding of the reasoning behind the decision-making mechanism and therefore revealing the importance of the considered country-level parameters in the COVID-19 spread throughout Europe. 

Table 3 cites the mean square error (MSE) achieved by the proposed ML pipeline for each one of the three pandemic waves. Comparable results are observed for waves 2 and 3 (MSE < 0.02), whereas a relatively higher MSE (~0.027) was reported for the case of wave 1. This difference in performance between wave 1 and waves 2–3 can be attributed to the fact that the data problems of waves 2 and 3 also include features from the previous wave (1 and 2, respectively), increasing the feature dimensionality of the data problems. The inclusion of new descriptive parameters enhances the predictive capacity of the ML model; however, it also poses a challenge, the so-called “curse of dimensionality”. The feature dimensionality problem was handled in our study with the use of ε-SVM, which is a well-known approach for its ability to handle high-dimensional spaces while keeping its generalization and high accuracy.

### 3.1. Wave 1 

**Figure 3:**
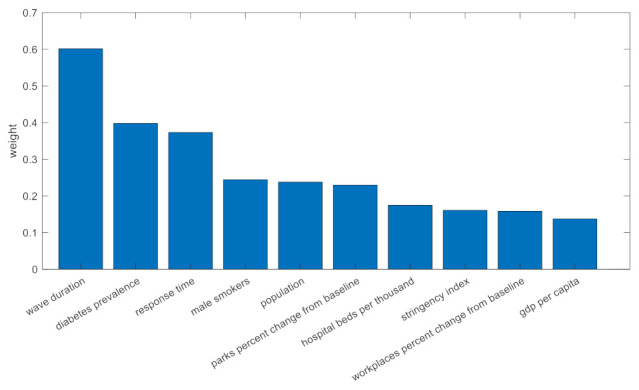
Impact of the selected risk factors for wave 1.

Figure 3 shows the most important contributing factors for wave 1 in descending order. As explained in Section 2.3, the impact of each parameter was quantified by its weight (|**w***_j_*|). In total, 10 parameters were proven to be important for the estimation of deaths in the first COVID-19 wave, with the total duration of the wave (in days) as the major contributor. Significance testing was also performed to assess whether a change in an input variable would change (or not) the predicted output of the model. *p*-Values were calculated on the basis of the t-statistic that was defined as the sample coefficient (**w***_j_*) divided by the standard error. All the factors depicted in Figure 3 were proven to be statistically significant at *p* < 0.05. 

**Figure 4:**
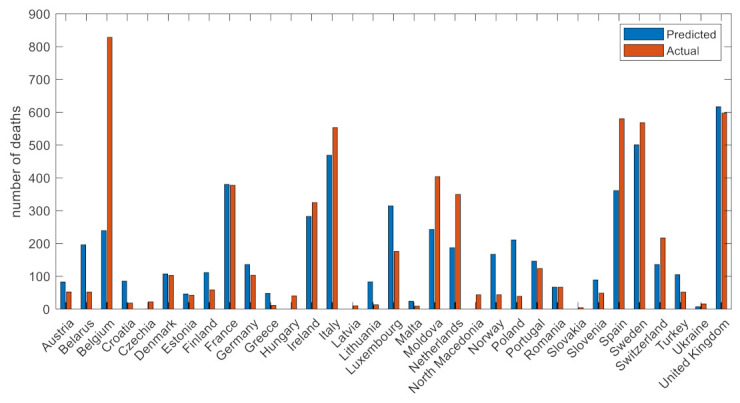
Actual versus predicted number of deaths in wave 1.

Figure 4 depicts the number of COVID-19 deaths in wave 1 with respect to the predicted number of deaths. The dashed grey line corresponds to the perfect scenario where the predicted number equals the actual number of deaths. Overall, the predictive performance of the ML pipeline was adequately high for the great majority of the countries with the extension of Belgium, which seems to be an outlier in the first phase of the COVID-19 pandemic. The most important factors in the first wave were the wave duration, comorbidities (i.e., diabetes prevalence), response time, smoking habits, and the population size as well as the mobility change and the capacity of the health care facilities. 

### 3.2. Wave 2 

**Figure 5:**
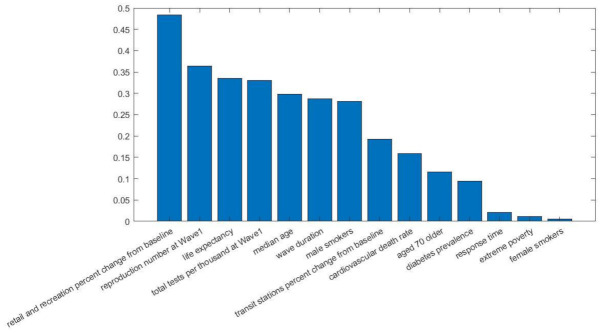
Impact of the selected risk factors for wave 2.

Figure 5 presents the most important risk factors that contributes to the prediction of deaths during the second wave. Fourteen parameters were finally selected, twelve extracted from wave 2 and another two from wave 1. The percentage mobility change in retail and recreating with respect to baseline was proven to be the most important factor. The most important factors in the second wave were the mobility change (populations’ response to public health measures), the effective reproduction number of wave 1, life expectancy, testing capacity, median age, smoking habits, cardiovascular death rate, proportion of people older than 70, and diabetes. Statistical analysis revealed that the variables response time, extreme poverty, and female smokers were not statistically significant, with reported *p*-values 0.341, 0.391, and 0.451, respectively. All the rest risk of the factors had significant contribution to the predicted output, with *p*-values lower than 0.015.

**Figure 6:**
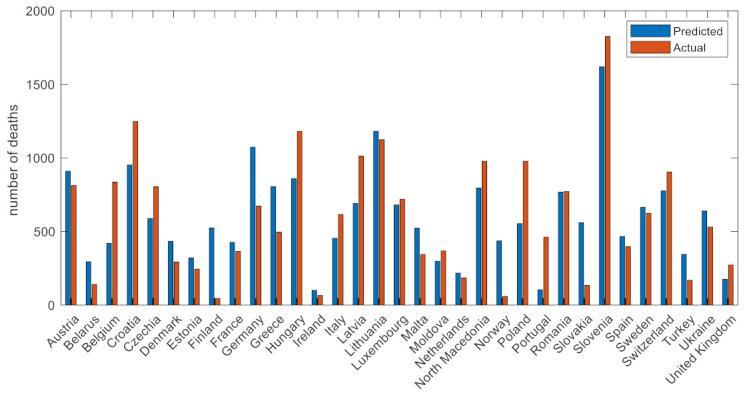
Actual versus predicted number of deaths in wave 2.

Figure 6 visualizes the predictive performance of the ML approach (with predicted versus actual number of deaths) for wave 2. The great majority of the predictions are within an accepted deviation range, demonstrating the predictive capacity of the model. 

### 3.3. Wave 3 

**Figure 7:**
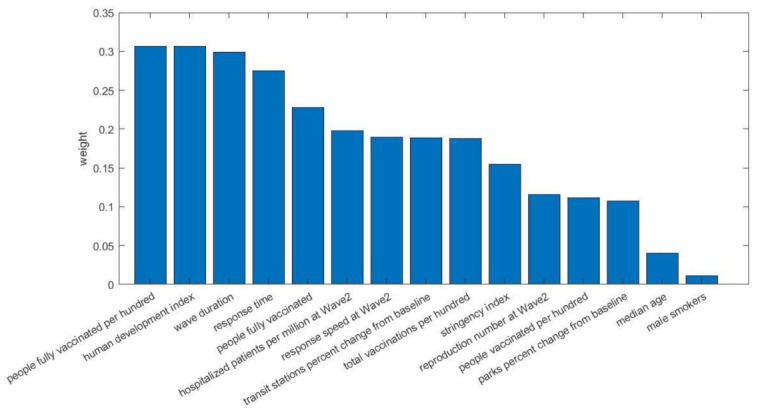
Impact of the selected risk factors for wave 3.

The most important contributing risk factors of wave 3 and their impact on the prediction of deaths are depicted in Figure 7. Fifteen risk factors were included in the model, with the number of fully vaccinated people (%) being the most important one. Another three risk factors relevant to the vaccination level of the EU countries were also selected (people fully vaccinated, total vaccinations per hundred, and people vaccinate per hundred), indicating the importance of vaccinations in general in the prediction of deaths. 

The most important factors in the third wave were vaccination coverage, human development index, wave duration, response time, number of hospitalized patients, response speed for wave 2, stringency index, mobility change, and effective reproduction number of wave 2. All variables were proven to be statistically significant at *p* < 0.05 expect median age and male smokers, which reported *p*-values of 0.111 and 0.417, respectively.

**Figure 8:**
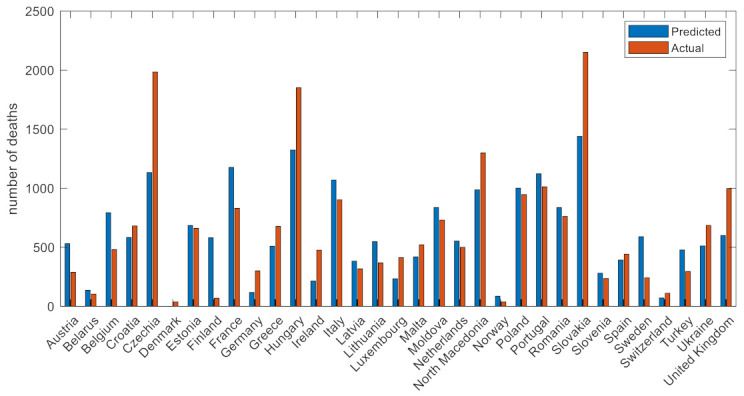
Actual versus predicted number of deaths in wave 3.

As seen in Figure 8, the performance of the predictive models for the majority of EU countries lies within an acceptance deviation margin (±20%). For three countries (Czechia, Slovakia, and Hungary), the actual number of deaths were relatively higher than the predicted number, whereas Finland had much lower deaths compared to the predicted number (~600) in wave 3.

## 4. Discussion

In this study, an ML pipeline was designed to estimate the number of deaths due to COVID-19 for each of the first three pandemic waves. However, the ultimate objective of the study is not the prediction of deaths but the identification of country-level risk factors that drove the COVID-19 pandemic course and outcomes in the EU countries. To achieve this, a hybrid dataset was created employing publicly available sources comprising heterogeneous parameters across the majority of European countries, e.g., mobility measures, policy responses, vaccinations, and demographics/generic country-level parameters. Data pre-processing and data exploration techniques were initially applied to normalize the available data and decrease the feature dimensionality of the data problem considered. Then, a linear ε-SVM model was employed to implement the regression task. The choice of linear ε-SVM was made due to the small sample size and based on the generalization capacity of SVΜs along with their ability to cope with high-dimensional spaces. This process was supported by an experimental evaluation per wave in which the results showed that the proposed ML pipeline achieved satisfactory results as reported in Table 2 (MSE of 0.027 for wave 1 and MSE less than 0.02 in waves 2 and 3). The post hoc analysis complemented the prediction findings by uncovering the rationale behind the decision-making mechanisms of the ML pipeline, thus enhancing our understanding with respect to the contribution of the selected country-level parameter to the prediction of deaths due to COVID-19 in the EU. 

Ten country-level parameters contributed significantly to the estimation of deaths in the first COVID-19 wave. Apart from total wave duration as the major contributor, diabetes prevalence and the country’s response time were also selected as the second and third most important factors. 

Mobility measures, such as percentage change in people’s mobility in transit stations and workplaces, also had an impact in the COVID-19-related mortality, whereas the stringency index was also an important contributor [30]. Demographics, such as population, GPD per capita, number of male smokers, and capacity of hospital beds per thousand, were also found to be significant factors. The first wave had unique characteristics due to the unknown nature of SARS-CoV-2 infection, peoples’ unawareness of the risks, the effectiveness of protective measures, the low capacity for testing, as well as the lack of experience in the clinical management of a new disease. Regarding Europe at the initial stage of the pandemic, the virus was spreading undetectably in the geographic areas of Italy and several other countries, thus suggesting that after entering the exponential phase, the ability to control the number of severe diseases and COVID-19-related deaths was limited. Due to these characteristics and the fact that our knowledge for the control and management of COVID-19 was limited, some of the critical factors for COVID-19 mortality were related to the intensity of the wave (i.e., the response time, wave duration), compliance to public health measures (change in mobility), and stringency of measures [31,32]. In countries of Western Europe, where public health measures were applied later with regard to the time of exponential phase imitation, the COVID-19-related mortality was much higher than in Eastern and Central Europe. Notably, some additional characteristics, such smoking and diabetes, which are known risk factors for severe COVID-19, were also associated with mortality. The joint effect of the aforementioned input parameters led to an overall good predictive performance in the majority of European countries. Belgium was an exception as seen in Figure 4, with the actual number of COVID-19 deaths being much higher than the expected one. This is in accordance with the epidemiological figures, where COVID-19 mortality in Belgium during that period was among the highest in Europe. 

The second wave started in autumn or early winter, and changes in mobility were indicated as the most significant risk factors during the second wave, especially in retail and recreation. Changes in mobility in transit stations were also found to have an impact. A number of demographics were also selected, such as life expectancy, median age of the population, cardiovascular death rate, number of people aged 70 or older, extreme poverty, and the number of female smokers. The second wave started as a result of human activities (i.e., changes in mobility) after a long period of very low viral circulation in the summer and early autumn 2020, which was due to a global lockdown after the first pandemic wave and climatological factors that do not favor virus spread during this period of the year. Age was one of the strongest prognostic factors for severe disease as picked up by the model (i.e., median age and number of people aged 70 or older) as well as cardiovascular disease [33]. Life expectancy is a measure of the quality of life and health care that is expected to reflect the capacity of health care system to respond to the increased hospitalization needs of the pandemic [34]. Two risk factors from the previous wave (wave 1) were also selected, specifically the effective reproduction number (R) and the total number of tests; the first parameter reflects the rate of the epidemic growth and the second the capacity of testing and thus of the ability to make timely diagnoses of cases and to quarantine their contacts. EU countries’ testing capacity combined with contact tracing have been identified as important health responses [35,36]; however, their efficacy deteriorated as the number of infections increased. Response time did not play as important a role as in the first wave.

Notably, four parameters associated with the vaccination progress status of the countries were selected in wave 3, with the number of the fully vaccinated people per hundred as the most important [30]. Having four vaccination-related metrics out of the fifteen selected features indicates the effect of the vaccination program on the number of COVID-19 deaths in EU during the third wave [37,38]. Stringency index and the response time were also found to contribute significantly to the prediction task. Similarly to the previous wave, the wave duration, mobility changes, and demographics were also proved to be related to the number of the deaths [34]. These factors reflect the intensity of the wave, the compliance to public measures, and risk factors for sever disease. Three factors from the previous wave (i.e., response speed, reproduction number, number of hospitalizations) did also contribute to the ML decision-making mechanism, suggesting that the intensity of the previous wave and therefore the acquired immunity from natural infection as well as the fear of COVID-19-related deaths as a result of the second wave may play a role in the control of a subsequent wave. Slovakia and Czechia had a different pandemic pattern, with the two waves not separating. For all these three countries, additional unknown factors were associated with COVID-19-related deaths. 

Regarding risk factors from previous waves and their effect on the prediction of mortality, effective reproduction number and number of tests at wave 1 were selected for the prediction of mortality in wave 2 since these parameters were associated with the intensity of the first wave. Geographic areas experienced large numbers of cases at the early phase of the pandemic were less affected by the second wave, probably due to a more immediate response. Similarly, the number of hospitalized patients, response time, and effective reproduction number at wave 2, which also provide a proxy of the intensity of the pandemic wave were selected for the prediction of mortality in wave 3 probably for the same reason as in previous waves. From our experience, the severity of a pandemic wave in a specific region was inversely correlated with the number of cases in the previous wave, suggesting that geographic areas with large number of cases were less affected in future waves.

Our study has some limitations. Firstly, a limitation of the study comes from the nature of linear regression models, which assumes the predicted output as a linear combination of weighted features. Each of the weights specifies the gradient of the linear hyperplane in one specific direction, isolating the interpretation of the associated input parameter from the rest. However, the joint contribution of the features is actually ignored given that the interpretation of a single feature always comes with the precondition that all other features remain unchanged. On the hand, the large size of the sample can be considered as a strength of the study.

Overall, the current study contributed to the identification of country-level risk factors that drove the COVID-19 pandemic in the EU countries via the use of an advanced ML methodology. The strength of the proposed approach lies on (i) the strong global search ability and optimization speed of the employed ε-SVM regression model, (ii) its known generalization capacity, (iii) its ability to accurately predict mortality on a small data sample, and (iv) the model’s transparency, which led us to better understand its inner workings and thus the impact of the input variables to mortality. The main findings of the study can be summarized as follows: Wave duration, mobility changes, and demographics were proven to be highly correlated with the number of deaths throughout the whole pandemic course so far (for all three waves considered). Diabetes prevalence and the countries’ response time were major contributors during the first wave, whereas changes in mobility and especially in retail and recreation were leading factors highly associated with mortality on the second wave. Finally, vaccination status of the countries played a significant role in wave 3, with the number of fully vaccinated people per hundred as the most significant risk factor. 

The application of more advanced explainability tools should be considered in future work. Graphical modeling combined with linear models will be employed to identify the direct and indirect effect of features to the prediction outcomes, providing a more intuitive, graphical way of interpreting the effect of country-level parameters on the spread of COVID-19 in Europe. 

## Figures and Tables

**Table 1:** Description of the feature categories in the employed dataset.

Category	Description
Confirmed cases	Demonstrates the new or total confirmed cases of SARS-CoV-2 (F33 in Table 2)
Confirmed deaths	Describes the COVID-19-related deaths (F34 and F40)
Hospital and intensive care units (ICU)	Describes variables which consists of data about the patients in hospital and the patients in intensive care units (F36 and F37)
Policy responses	Government Response Stringency Index, which is composite measure based on 9 response indicators (0 to 100, 100 = strictest response) (F7 and F8)
Reproduction number	Real-time estimate of the effective reproduction number (R) of COVID-19 (F35)
Tests and positivity	Consists of variables which demonstrate information about the total number of tests per 1000, new tests per 1000, and the tests that are positive given as a rolling 7-day average (F38–39)
Vaccinations	Information about the vaccination doses and the booster doses that have been administered (F9–17)
Mobility	Includes mobility trends for places such as markets, drug stores, public areas, transport hubs, retail, recreation, places of residence, and workplaces (F1–6)
Generic	Includes variables that describe demographic data and data that occur from the quality of life (F18–32)

Government Response Stringency Index is a composite score, which is based on nine response indicators, including workplace closures, school closures, and travel bans.

**Table 2:** Description of the features extracted per wave per country.

#	Category	Description		Current Wave	Previous Wave
F1	Mobility	Grocery and pharmacy percent change from baseline	Mean	✔	
F2		Parks percent change from baseline		✔	
F3		Residential percent change from baseline		✔	
F4		Retail and recreation percent change from baseline		✔	
F5		Transit stations percent change from baseline		✔	
F6		Workplaces percent change from baseline		✔	
F7	Policy responses	Stringency index		✔	✔
F8		Response time	See (1)	✔	✔
F9	Vaccinations	Total vaccinations (cumulative)	Last valid	✔	
F10		People vaccinated (cumulative)		✔	
F11		People fully vaccinated (cumulative)		✔	
F12		New vaccinations	Mean	✔	
F13		New vaccinations smoothed		✔	
F14		Total vaccinations per hundred (cumulative)	Last valid	✔	✔
F15		People vaccinated per hundred (cumulative)		✔	✔
F16		People fully vaccinated per hundred (cumulative)		✔	✔
F17		New vaccinations (smoothed) per million	Mean	✔	
F18	Demographics	Population	Mean	✔	
F19		Population density		✔	
F20		Median age		✔	
F21		Aged 65 older		✔	
F22		Aged 70 older		✔	
F23		GDP per capita		✔	
F24		Extreme poverty		✔	
F25		Cardiovasc death rate		✔	
F26		Diabetes prevalence		✔	
F27		Female smokers		✔	
F28		Male smokers		✔	
F29		Handwashing facilities		✔	
F30		Hospital beds per thousand		✔	
F31		Life expectancy		✔	
F32		Human development index		✔	
F33	Cases, deaths, hospitalizations, and positivity	Total cases per million	Last valid		✔
F34		Total deaths per million			✔
F35		Reproduction number	Mean		✔
F36		ICU patients per million (cumulative)	Last valid		✔
F37		Hospitalized patients per million (cumulative)			✔
F38		Total tests per thousand (cumulative)			✔
F39		Positive rate given as a rolling 7-day average	Mean		✔
F40		Total deaths per million in the wave (cumulative)	Last valid		✔

**Table 3:** Predictive performance achieved by the proposed ML pipeline for each of the three waves.

Metric	Wave 1	Wave 2	Wave 3
Mean square error ^1^	0.02707	0.01829	0.01913

^1^ MSE was calculated on the normalized data to set a fair basis of comparison between the waves.

## Data Availability

Data are accessible on the databases “Our World in Data” (https://ourworldindata.org/coronavirus, access on 8 October 2021) [39] and “Google COVID-19 Community Mobility Reports” (https://www.google.com/covid19/mobility/, access on 8 October 2021) [40].
